# Reversibility of oxalate nephropathy in a kidney transplant recipient with prior gastric bypass surgery

**DOI:** 10.1093/ckj/sfaa254

**Published:** 2021-01-04

**Authors:** Christian Goul Sørensen, Christian Lodberg Hvas, Ingrid Møller Thomsen, Bente Jespersen

**Affiliations:** 1 Department of Renal Medicine, Aarhus University Hospital, Aarhus, Denmark; 2 Department of Hepatology and Gastroenterology, Aarhus University Hospital, Aarhus, Denmark; 3 Nephrology and Hypertension University Clinic, Hospital Unit West, Holstebro, Denmark

**Keywords:** bile acid binders, gastric bypass, haemodialysis, hyperoxaluria, kidney transplantation, oxalate nephropathy, Roux en-Y gastric bypass, secondary oxalosis

## Abstract

Bariatric surgery is an acknowledged treatment for obesity and related comorbidities with beneficial effects on kidney function. However, bariatric surgery can also lead to secondary hyperoxaluria and oxalate nephropathy, resulting in end-stage kidney disease in both native and transplanted kidneys. We present a 66-year-old man who was in need of dialysis 3 months after kidney transplantation due to recurrent oxalate nephropathy. Intensified haemodialysis together with increased liquid intake, dietary restrictions of oxalate and fat and supplementation with calcium citrate and a bile acid binder were applied. Graft function improved and the patient did not require dialysis during the following 8 months.

## BACKGROUND

Bariatric surgery induces sustained weight loss and a decrease in obesity-associated comorbidities, including hypertension and diabetes mellitus, with positive effects on kidney function [1]. Set against the benefits to kidney function is the risk that secondary hyperoxaluria and oxalosis may develop, more frequently following malabsorptive procedures such as Roux-en-Y gastric bypass (RYGB) than restrictive procedures such as gastric banding or sleeve gastrectomy [2]. Hyperoxaluria may cause nephrolithiasis, acute kidney injury, oxalate nephropathy and end-stage kidney disease (ESKD) [1] in both native and transplanted kidneys [3]. We describe a case of post-transplant oxalate nephropathy and its management.

## CASE REPORT

A 60-year-old male underwent RYGB because of a body mass index (BMI) of 43 kg/m^2^, type 2 diabetes, hypertension and sleep apnoea. Before surgery his plasma creatine level was 104–143 µM with <30 mg albumin/g creatinine in urine. Surgery caused a 50-kg weight loss, a BMI of 26 kg/m^2^ and remission of diabetes, hypertension and sleep apnoea.

**FIGURE 1:**
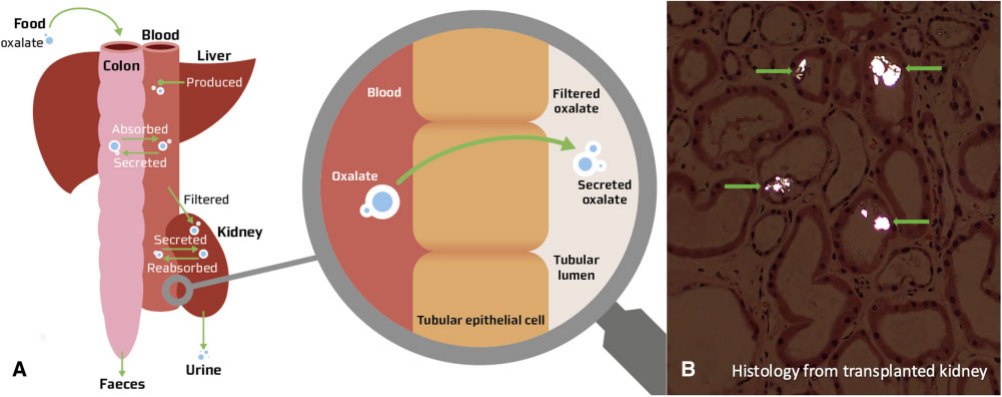
(**A**) Oxalate metabolism during normal circumstances: dietary intake, colonic formation of calcium-oxalate crystals, passive absorption of free oxalate in the colon and renal excretion through filtration and active tubular oxalate secretion in the normal kidney. (B) Histology from the transplanted kidney with oxalate deposition (arrows).

One year after surgery the patient’s renal function gradually declined and a biopsy revealed oxalate nephropathy with 24-h urine oxalate on 1469 μmol. Standard advice to increase liquid intake and eat less fat, vitamin C and oxalate-containing food items combined with oral calcium supplementation were insufficient to decrease plasma and urine oxalate below precipitating levels. The patient reported compliance with diet advice, but this was not monitored. He started haemodialysis and underwent his first renal allograft transplantation but lost the graft due to post-surgical bleeding and thrombosis. Two years later he had a second kidney transplantation with an uneventful recovery and an estimated glomerular filtration rate (eGFR) of 53 mL/min. However, 2 months post-transplantation, kidney function deteriorated and a renal biopsy confirmed inflammation and oxalate crystal deposition (Figure 1). Urinary oxalate was 711 μmol/24 h and serum oxalate was 68.7 μmol/L. When starting dialysis, creatinine clearance was only 2 mL/min and 24-h diuresis was 700 mL. At this point, intensive haemodialysis up to 4 h daily was initiated with stricter diet restrictions, increased fluid intake, calcium citrate supplementation plus a bile acid sequestrant in the form of cholestyramine. The patient gained sufficient kidney function to reduce and finally stop dialysis when he was able to eliminate oxalate renally to keep plasma oxalate <30 µM. He stayed out of dialysis for the following 8 months.

## DISCUSSION

This case report shows that oxalate nephropathy may complicate bariatric surgery. It further illustrates the potential reversibility of the condition using several treatments and lifestyle changes [4].

Oxalate is found in plant-based food items, including green leafy vegetables, seeds, cocoa and tea, and is endogenously produced during hepatic glyoxylate metabolism [5]. Ingested vitamin C metabolizes into oxalate [3]. Under normal circumstances, orally consumed calcium and oxalate form complexes within the colonic lumen and are excreted in the faeces. Free oxalate is absorbed passively and excreted in the urine. Following RYGB, the reduction in functional small bowel surface area results in increased amounts of fatty acids in the colon. Because fatty acids preferentially bind calcium, this reduces the formation of calcium–oxalate complexes [5]. Bile salts and mucosal inflammation may further increase intestinal permeability, leading to absorption of even greater amounts of soluble oxalate [2]. From the bloodstream, oxalate is eliminated through glomerular filtration and tubular secretion [5], and high levels cause hyperoxaluria and formation of oxalate crystals and kidney stones [2]. Hyperoxaluria develops in up to two-thirds of patients in the first year following RYGB [2]. As with our patient, oxalate nephropathy can occur without the formation of kidney stones and the frequency may be underreported [3].

Comorbidities, including chronic kidney disease, facilitate oxalate nephropathy partly due to impaired oxalate excretion [5]. Genetic variants may predispose to oxalate accumulation [3], but this was not suspected in our 60-year-old patient. A high proportion of oxalate nephropathies leads to ESKD [3]. Oxalate nephropathy can be prevented and treated and is to some extent reversible. Our patient regained kidney graft function with intensive haemodialysis and multiple interventions. The use of additional bile acid sequestrants may have contributed to the reduction of secondary oxalosis by binding bile acids, reducing the mucosal permeability and binding of free oxalate [4]. Experimental therapies involving *Oxalobacter formigenes* colonization to further decrease hyperoxaluria are being evaluated [4, 5]. After transplantation, early graft function is pivotal for renal oxalate clearance, and supplementary dialysis is often needed. It is well known that a GFR >30 mL/min and high fluid intakes are necessary to eliminate high loads of oxalate in both primary and secondary oxalosis [5].

In conclusion, our case report illustrates the importance of acknowledging oxalate nephropathy as a complication of bariatric surgery. In patients with secondary oxalosis, several treatments are available and these may help to conserve native and transplant kidney function. We suggest that the risk for oxalate nephropathy and subsequent ESKD should be considered when advising bariatric surgery for obesity.

## PATIENT CONSENT

Informed consent was obtained from the patient as specified in the International Committee of Medical Journal Editors recommendations.
